# Socio-demographic, ecological factors and dengue infection trends in Australia

**DOI:** 10.1371/journal.pone.0185551

**Published:** 2017-10-02

**Authors:** Rokeya Akter, Suchithra Naish, Wenbiao Hu, Shilu Tong

**Affiliations:** 1 School of Public Health and Social Work, Institute of Health & Biomedical Innovation, Queensland University of Technology, Brisbane, Queensland, Australia; 2 Shanghai Children's Medical Centre, Shanghai Jiao Tong University, Shanghai, China; 3 School of Public Health, Anhui Medical University, Hefei, China; Institut Pasteur, FRANCE

## Abstract

Dengue has been a major public health concern in Australia. This study has explored the spatio-temporal trends of dengue and potential socio- demographic and ecological determinants in Australia. Data on dengue cases, socio-demographic, climatic and land use types for the period January 1999 to December 2010 were collected from Australian National Notifiable Diseases Surveillance System, Australian Bureau of Statistics, Australian Bureau of Meteorology, and Australian Bureau of Agricultural and Resource Economics and Sciences, respectively. Descriptive and linear regression analyses were performed to observe the spatio-temporal trends of dengue, socio-demographic and ecological factors in Australia. A total of 5,853 dengue cases (both local and overseas acquired) were recorded across Australia between January 1999 and December 2010. Most the cases (53.0%) were reported from Queensland, followed by New South Wales (16.5%). Dengue outbreak was highest (54.2%) during 2008–2010. A highest percentage of overseas arrivals (29.9%), households having rainwater tanks (33.9%), Indigenous population (27.2%), separate houses (26.5%), terrace house types (26.9%) and economically advantage people (42.8%) were also observed during 2008–2010. Regression analyses demonstrate that there was an increasing trend of dengue incidence, potential socio-ecological factors such as overseas arrivals, number of households having rainwater tanks, housing types and land use types (e.g. intensive uses and production from dryland agriculture). Spatial variation of socio-demographic factors was also observed in this study. In near future, significant increase of temperature was also projected across Australia. The projected increased temperature as well as increased socio-ecological trend may pose a future threat to the local transmission of dengue in other parts of Australia if *Aedes* mosquitoes are being established. Therefore, upgraded mosquito and disease surveillance at different ports should be in place to reduce the chance of mosquitoes and dengue cases being imported into all over Australia.

## Introduction

Dengue is a significant health issue for mankind mostly in tropical and subtropical regions of the world [1]. Globally, dengue caused 390 million infections annually in more than 125 countries [2]. The severity of dengue incidence has increased 30 fold in the past 50 years [1] and distribution has been expanded to previously unoccupied and less warm countries, for example, European countries [3].

Dengue transmission dynamics are complex, involving virus, vector and host. Four closely related dengue viruses under the family flaviridae are responsible for dengue transmission. Dengue virus is transmitted between humans by the domesticated and fresh water breeding mosquito species, *Aedes aegypti* (primary vector) and *Aedes albopictus* (secondary vector) [4]. All of these parameters (virus, vector and human host) are influenced by climatic (temperature, rainfall and relative humidity), human-related, socio-economic, demographic and ecological factors [5, 6]. Climate, especially, temperature accelerates the mosquito bite, development rates, mortality, and behaviour and control the reproductive capacity of the virus within the mosquito [7, 8]. Climate also brings changes in human behaviour and life style which in turn influence the dengue transmission dynamics. For example, increased urban water hoarding in response to climate change (e.g., decreased rainfall or drought) can increase the number of productive larval sites if provisions are not made to eliminate this risk. As a result, even small amount of rain may lead to increase of A. *aegypti* densities [9].

In Australia, dengue was first introduced by ship from Mauritius in 1873 [10]. The first local outbreaks occurred at Townsville and Rockhampton in Queensland in 1879 and 1885, respectively. Historically, dengue was present in Western Australia, New South Wales and Northern Territory during late nineteenth century and early twentieth century. However, epidemic outbreaks were reported during 1925–1926 in New South Wales and distribution was extended towards southward including Newcastle. Reticulated water supply instead of rainwater tanks has been considered as one of the reasons behind the disappearance of local transmission of dengue from New South Wales, Western Australia, Northern Territory and Queensland [10]. Dengue re-emerged in northern Queensland during 1981–1982 [10] and since then dengue has been transmitted locally whereas in other states, it is overseas-acquired. Several researchers have studied the future distribution of *Aedes* mosquitos under future climate change scenarios in Australia and suggested that dengue distribution could expand to the west and south in coming decades [11], while some others have predicted that the disease could expand to other regions and even throughout the country [12, 13]. A recent study predicted that future distribution of *Aedes* mosquito could expand to East coast of Australia including Queensland, Northern Territory, Australian Capital Territory and New South Wales [14].

Evidence from the literature indicates a significant involvement of socio-demographic and ecological factors in dengue transmission around the world [15] including: climate especially temperature [16–20] and rainfall [18]; urbanisation [6, 21–24]; movement of vectors and hosts via travel [6, 21–24]; land use or land cover change [25– 29]; house types or density [30, 31]; population density [6, 21, 22]; Indigenous population [32]; socio-economic conditions [17, 33–35] and water storage behaviour [9]. Climate change and globalisation through improved transportation and human movements will favour the distribution of vector mosquito and dengue virus to other parts of Australia. Human behaviour and life style as well as installation of increased number of rainwater tanks could provide favourable conditions for mosquitoes to habitat and breed and hence could increase the chance of biting. Availability of potential socio-demographic and ecological factors could lead to initiatiation of local transmission of dengue through establishment of mosquito population to other parts of Australia due to increasing trend of transportation and globalisation. In recent times, establishment of *A*. *albopictus* in Torres Strait Island [36, 37] as well as several incursions of *A*. *albopictus* in different ports such as Cairns, Brisbane, Townsville, Sydney, Darwin and Melbourne has exacerbated the risk of establishment of this mosquito in mainland Australia. Even though, dengue is not endemic in Austrlia and the number of cases are very low compared to dengue endemic countries in Asia-Pacific region, it should be noted that under favourable climatic and socio-ecological conditions as well as in the possible establishment of *A*. *albopictus* within Australia, this number could be beyond our control like in USA [38]. Therefore, it is necessary to explore the recent trend and spatio-temporal distribution of potential socio- demographic and ecological factors on dengue infection in Australia to understand the future severity of dengue risk to undertake preventive control measures. Hence, in this paper we aimed to evaluate the epidemiology of dengue, assess the trend of potential socio-demographic, ecological predictors, and explore the future vulnerability of dengue in Australia.

## Materials and methods

### Study area

Australia, officially known as the Commonwealth of Australia [39], is an Island Continent. It is located south of the Equator and bounded by the Indian Ocean to the west and Pacific Ocean in the east. It is the world's sixth-largest country by total area. Neighbouring countries include Papua New Guinea, Indonesia and East Timor to the north; the Solomon Islands and Vanuatu to the north-east; and New Zealand to the south-east. It occupies cool temperate to tropical climates from 10° to 43° south latitudes and from 112° to 153° east longitudes. Australia is comprised of 6 states: New South Wales (NSW), South Australia (SA), Queensland (QLD), Western Australia (WA), Victoria (VIC) and Tasmania (TAS), and 2 territories: The Australian Capital Territory (ACT) and the Northern Territory (NT). It consists of four typical seasons in a year: summer: December to February; autumn: March to May; winter: June to August and spring: September to November.

### Data collection

#### Dengue data

Dengue is a nationally notifiable disease in Australia since 1993 [40]. A confirmed case requires clinical evidence and laboratory confirmation. Laboratory methods include virus isolation, nucleic acid testing, detection of dengue non-structural protein 1 (NS1) antigen, dengue virus-specific IgG seroconversion. All the laboratory-confirmed cases are required to be reported to health departments within each state and territory under Public Health Act 2005. Dengue cases at state level were obtained from Australia’s National Notifiable Disease Surveillance Systems (NNDSS) for the period January 1999 to December 2010 (11 years). This study period was chosen due to the availability of all the datasets. Both overseas-acquired and locally-transmitted cases of dengue were documented in Queensland whereas in other regions, it was only overseas-acquired. However, local transmission of dengue in Queensland often initiates by *Aedes* mosquito bites of infected travellers [41].

#### Climate data

Annual mean minimum, maximum temperature and mean rainfall for all capital cities of Australia for the study period were obtained from the Australian Bureau of Meteorology. Then mean temperature was calculated. Capital cities were selected as representative of the entire state as most of the population lives in and around the capital cities. Projection of the climatic conditions under Representative Concentration Pathways (RCP) 4.5 for 2020–2039 was also obtained from Climate change in Australia website (https://www.climatechangeinaustralia.gov.au/en/climate-projections/explore-data/data-download/station-data-download/)

#### Socio-demographic data

Socio-demographic data including population (cat. no. 3218.0), income, housing structure, Indigenous status (cat. no. 4705.0) at state level for the national census years 2001, 2006 and 2011were obtained from Australian Bureau of Statistics (ABS) [42]. Income and housing structure for each state were gathered from ABS through community profiles, which give time series statistics for the census years. Data on number of households having rain water tank for each state for the study period 1999–2010 were obtained from ABS, Environmental issues: People’s view and Practices, 1998, 2001, 2004, 2010 (cat. no. 4602). This is a publication of ABS where households were surveyed and the households having water tank data were analysed. For the remaining years, data were estimated using linear interpolation. Overseas visitors (cat no. 3105.0.65.001.) and interstate arrival data for the period 1999–2010 were also obtained from ABS.

#### Land use raster data and data processing

Recent evidence suggested that land use types are associated with dengue [17, 43–46]. Different types of agricultural land use, water bodies and forest other than residential areas are found to be associated with reported dengue [28]. Therefore, it is necessary to consider land use types in relation to dengue. Among the three land use types in Australia, we focused on a broad category such as primary land use types to draw an overall picture of dengue and land use trends in Australia.

Land use raster data for the period of 1998–2011 were obtained from the Australian Bureau of Agricultural and Resource Economics and Sciences (ABARES) (http://www.agriculture.gov.au/abares/aclump/land-use/). To obtain the percentage of land use types at different states of Australia, a vector data file, based on Australian Geographic Classification 2011, was obtained from Australian Bureau of Statistics. To ensure the spatial alignment, both vector and land use raster data were projected into GDA_1994_Albers. Then Tabulate Intersection Tool in ArcGIS (version 10.2, 2013) was used to get the percentage of each land use types by states. Land use maps are not available for each year. Therefore, 2001–2002 land use data was used as representative for the period of 2002–2004.

### Data analysis

State-wide dengue, socio-demographic and ecological data were analysed using frequencies for the entire study period (1999–2010) and by sub periods (1999–2001, 2002–2004, 2005–2007 and 2008–2010) for the ease of investigation of spatial and temporal patterns of dengue and potential socio-ecological factors linked to dengue. For temporal trend analysis, linear regression line was fitted. State-wide incidence rates of dengue were calculated within each period as following: = (total number of dengue cases/total person-years)*100,000 and then mapped.

For mapping purposes, state-wide socio-demographic factors were calculated as following: (total number of the factors/ total person-years)*100. Additionally, households having rainwater tanks, the denominator was the total number of households rather than total person-years. All the analyses were performed using SPSS (version 22, 2013) and Excel software. ArcGIS (version 10.2, 2013) was used for visualisation of the socio-demographic and ecological factors.

#### Ethical considerations

The study was approved by the Human Research Ethics committee, Queensland Health Data Custodian under the Public Health Act, 2005 followed by Research Ethics Unit, Queensland University of Technology (approval number: 1500001029).

## Results

### Descriptive statistics

During the study period, a total of 5,853 dengue cases were reported in Australia with the highest number of dengue cases in QLD (n = 3,118) followed by NSW (n = 968). The highest number of interstate arrivals was recorded in QLD (n = 1,219,579) whereas NSW had higher overseas arrivals (n = 49,776,691). Both NSW and QLD had nearly the same number of households with rainwater tanks (n = 4,325.6 and 4,390.7, respectively). Highest number of Indigenous people were recorded in NSW (n = 1,623,888) followed by QLD (n = 1,505,293.2). NSW has the highest number of separate houses (n = 20,351,805) and terrace houses (2,838,154). Most of the people with weekly income above AUD$2500 lived in NSW (n = 1,612,305), followed by VIC (n = 1,065,687.02) (Table 1).

**Table 1:** Descriptive statistics of dengue and its potential socio-demographic factors across different states/territories of Australia during 1999–2010.

Variables	State/ Territory	Mean	Max	Range	Total	Variables	Mean	Max	Range	Total
**Dengue cases**	NSW	80.66	234	222	968	**Separate house**	1695984.00	1763180.00	144658.00	20351805.00
	VIC	20.66	121	121	248		1411616.60	1521420.40	202506.60	16939399.20
	QLD	259.83	1025	983	3118		1121485.53	1241155.00	239724.40	13457826.40
	SA	12.83	32	28	154		473751.47	503883.00	60507.60	5685017.60
	WA	75.41	504	497	905		585245.57	654700.00	131874.80	7022946.80
	TAS	1.91	7	7	23		161300.67	169960.80	16458.80	1935608.00
	NT	32.25	94	80	387		42044.00	44504.00	5389.00	504528.00
	ACT	6.66	18	17	80		90857.23	95846.20	9886.00	1090286.80
**Interstate arrivals**	NSW	88578.08	97189	16830	1062937	**Terrace house**	236513.00	270442.00	61788.00	2838154.00
	VIC	68494.08	74029	11532	821929		163069.03	190867.20	58392.60	1956828.40
	QLD	101631.6	119551	34818	1219579		107428.57	132632.80	49244.60	1289142.80
	SA	25668.67	30016	9161	308024		66472.15	68490.40	3746.40	797665.80
	WA	32987.58	38905	9564	395851		81138.40	88296.20	9116.00	973660.80
	TAS	13179.17	16261	4563	158150		8818.75	10259.00	2400.00	105825.00
	NT	16117.08	17331	2456	193405		6664.25	7525.40	1157.40	79971.00
	ACT	19100.75	20689	2618	229209		16346.50	19051.60	4774.60	196158.00
**Overseas arrivals**	NSW	4148058	6536351	3394196	49776691	**Number of households having rainwater tank (‘000**)	360.46	509.00	273.00	4325.60
	VIC	2077952	2909344	1434114	24935421		353.80	632.00	390.63	4245.70
	QLD	2250976	2714577	931659	27011707		365.89	633.00	398.30	4390.70
	SA	351656	491284	239887	4219872		319.87	343.80	38.80	3838.50
	WA	1103911	1646115	805721	13246927		98.68	129.80	59.17	1184.20
	TAS	79579.92	111572	58677	954959		39.78	48.40	16.73	477.40
	NT	100224.3	127827	48225	1202692		3.00	5.70	5.00	36.10
	ACT	139635.8	195649	103647	1675630		7.53	20.20	18.43	90.40
**Indigenous population**	NSW	135324.00	165487.60	52951.00	1623888.00	**People with weekly income above AUD$2500**	134359.00	254355.00	236661.00	1612305.00
	VIC	29102.13	36478.40	13430.00	349225.60		88807.25	174418.80	155128.17	1065687.02
	QLD	125441.1	150381.8	43559	1505293.2		83834.97	128748.40	61166.40	1006019.62
	SA	25375.80	29444.20	6876.80	304509.60		19045.57	40351.20	39696.00	228546.80
	WA	60470.17	67652.2	9238.2	725642		38510.19	88555.00	88534.31	462122.31
	TAS	16707.93	18875.4	3457.6	200495.2		4237.38	9313.80	9003.84	50848.51
	NT	52923.47	56152.8	6623.4	635081.6		2945.49	6529.80	6419.84	35345.88
	ACT	3995.80	4984.20	1563.80	47949.60		11397.50	22129.40	21126.40	136770.00

Max, Maximum

Among four different time periods, 2008–2010 period had the highest percentage of dengue cases (n = 3190.00, 54.22%). Similarly, socio-demographic factors such as overseas arrivals (n = 36894188.00, 29.99%), households having rainwater tanks (n = 6302.60, 33.91%), Indigenous populations, (n = 1532379.60, 28.42%), terrace house types (n = 2298925.20, 27.91%) and economically advantage peoples (1949668.20, 42.79%) had its highest percentage during this period (Table 2).

**Table 2:** Descriptive statistics of dengue and its potential socio-demographic factors across different states/territories of Australia during four different time periods.

Variables	State /Territory	Time period									
		1999–01		2002–04		2005–07		2008–10		Total	
		n	%	n	%	n	%	n	%	N	%
**Dengue cases**	**NSW**	89.0	16.8	171.0	11.9	182.0	25.2	526.0	16.5	968.0	16.5
	**VIC**	9.0	1.7	34.0	2.4	36.0	5.0	169.0	5.3	248.0	4.2
	**QLD**	188.0	35.5	1083.0	75.1	311.0	43.1	1536.0	48.2	3118.0	53.0
	**SA**	20.0	3.8	21.0	1.5	39.0	5.4	74.0	2.3	154.0	2.6
	**WA**	40.0	7.6	42.0	2.9	89.0	12.3	734.0	23.0	905.0	15.4
	**TAS**	2.0	0.4	3.0	0.2	3.0	0.4	15.0	0.5	23.0	0.4
	**NT**	168.0	31.7	72.0	5.0	50.0	6.9	97.0	3.0	387.0	6.6
	**ACT**	14.0	2.6	16.0	1.1	11.0	1.5	39.0	1.2	80.0	1.4
	**Australia**	530.0	9.0	1442.0	24.5	721.0	12.3	**3190.0**	**54.2**	5883.0	100.0
**Interstate**	**NSW**	281536.0	25.4	277403.0	23.7	247829.0	23.4	256169.0	24.3	1062937.0	24.2
	**VIC**	211855.0	19.2	219175.0	18.8	193346.0	18.2	197553.0	18.8	821929.0	18.7
	**QLD**	291564.0	26.4	344126.0	29.4	304179.0	28.7	279710.0	26.6	1219579.0	27.8
	**SA**	85123.0	7.7	86250.0	7.4	71452.0	6.7	65199.0	6.2	308024.0	7.0
	**WA**	92670.0	8.4	93377.0	8.0	99115.0	9.4	110689.0	10.5	395851.0	9.0
	**TAS**	36676.0	3.3	45285.0	3.9	37914.0	3.6	38275.0	3.6	158150.0	3.6
	**NT**	48623.0	4.4	45595.0	3.9	49241.0	4.6	49946.0	4.7	193405.0	4.4
	**ACT**	58464.0	5.3	57769.0	4.9	57329.0	5.4	55647.0	5.3	229209.0	5.2
	**Australia**	1106511.0	25.2	1168980.0	26.6	1060405.0	24.2	1053188.0	24.0	4389084.0	100.0
**Overseas arrivals ('000)**	**NSW**	13072.7	47.2	10427.7	40.0	12544.3	38.7	13732.0	37.2	49776691.0	40.5
	**VIC**	4740.4	17.1	5301.2	20.3	6802091.0	21.0	8091737.0	21.9	24935421.0	20.3
	**QLD**	5613426.0	20.3	5944747.0	22.8	7468536.0	23.1	7984998.0	21.6	27011707.0	22.0
	**SA**	835533.0	3.0	853505.0	3.3	1152471.0	3.6	1378363.0	3.7	4219872.0	3.4
	**WA**	2630844.0	9.5	2718264.0	10.4	3416382.0	10.6	4481437.0	12.2	13246927.0	10.8
	**TAS**	175865.0	0.6	203377.0	0.8	258153.0	0.8	317564.0	0.9	954959.0	0.8
	**NT**	294210.0	1.1	258859.0	1.0	288140.0	0.9	361483.0	1.0	1202692.0	1.0
	**ACT**	315168.0	1.1	366445.0	1.4	447457.0	1.4	546560.0	1.5	1675630.0	1.4
	**Australia**	27678104.0	22.5	26074091.0	21.2	32377516.0	26.3	**36894188.0**	**30.0**	123023899.0	100.0
**Households having rain water tank ('000)**	**NSW**	760.7	21.8	895.0	22.2	1211.1	25.5	1458.8	23.2	4325.6	23.3
	**VIC**	729.1	20.9	855.5	21.2	1033.2	21.7	1627.9	25.8	4245.7	22.8
	**QLD**	717.7	20.6	966.0	23.9	1081.0	22.7	1626.0	25.8	4390.7	23.6
	**SA**	955.2	27.4	928.6	23.0	946.4	19.9	1008.3	16.0	3838.5	20.7
	**WA**	220.4	6.3	264.9	6.6	324.3	6.8	374.6	5.9	1184.2	6.4
	**TAS**	96.7	2.8	110.2	2.7	128.7	2.7	141.8	2.3	477.4	2.6
	**NT**	4.2	0.1	6.5	0.2	10.3	0.2	15.1	0.2	36.1	0.2
	**ACT**	6.4	0.2	10.5	0.3	23.4	0.5	50.1	0.8	90.4	0.5
	**Australia**	3490.4	18.8	4037.2	21.7	4758.4	25.6	**6302.6**	**33.9**	18588.6	100.0
**Indigenous population**	**NSW**	348602.4	29.0	381580.2	29.7	417719.2	30.4	475986.6	31.3	1623888.4	30.2
	**VIC**	72189.6	6.0	81322.8	6.3	91022.8	6.6	104690.4	6.9	349225.6	6.5
	**QLD**	329392.2	27.4	356163.6	27.8	385644.6	28.1	434092.8	28.6	1505293.2	28.0
	**SA**	68988.6	5.7	72847.8	5.7	77247.0	5.6	85426.2	5.6	304509.6	5.7
	**WA**	175365.0	14.6	175734.0	13.7	178299.8	13.0	196243.2	12.9	725642.0	13.5
	**TAS**	46786.2	3.9	48384.6	3.8	50359.0	3.7	54965.4	3.6	200495.2	3.7
	**NT**	150471.6	12.5	156121.8	12.2	161701.4	11.8	166786.8	11.0	635081.6	11.8
	**ACT**	10494.6	0.9	11194.8	0.9	12072.0	0.9	14188.2	0.9	47949.6	0.9
	**Australia**	1202290.2	22.3	1283349.6	23.8	1374065.8	25.5	**1532379.6**	**28.4**	5392085.2	100.0
**Separate house**	**NSW**	4899817.0	31.1	5032573.0	30.7	5160928.0	30.3	5258487.0	29.6	20351805.0	30.4
	**VIC**	4003975.2	25.4	4145677.0	25.3	4294707.0	25.2	4495040.0	25.3	16939399.2	25.3
	**QLD**	3069851.4	19.5	3266530.0	19.9	3463044.0	20.3	3658401.0	20.6	13457826.4	20.1
	**SA**	1346743.0	8.5	1396592.0	8.5	1446336.0	8.5	1495347.0	8.4	5685018.0	8.5
	**WA**	1601153.0	10.2	1699184.0	10.4	1800231.0	10.6	1922379.0	10.8	7022947.0	10.5
	**TAS**	464592.0	3.0	476850.0	2.9	489477.2	2.9	504688.8	2.8	1935608.0	2.9
	**NT**	119034.0	0.8	124101.0	0.8	128967.0	0.8	132426.0	0.8	504528.0	0.8
	**ACT**	260533.8	1.7	268493.4	1.6	276492.4	1.6	284767.2	1.6	1090286.8	1.6
	**Australia**	15765699.4	23.5	16410000.4	24.5	17060182.6	25.5	17751536.0	26.5	66987418.4	100.0
**Terrace house**	**NSW**	639974.4	34.7	682015.2	34.4	726657.2	34.4	789507.6	34.3	2838154.4	34.5
	**VIC**	414656.4	22.5	466354.2	23.5	516853.6	24.4	558964.2	24.3	1956828.4	23.8
	**QLD**	263050.8	14.3	301709.4	15.2	340866.8	16.1	383515.8	16.7	1289142.8	15.7
	**SA**	199081.8	10.8	200101.8	10.1	195820.8	9.3	202661.4	8.8	797665.8	9.7
	**WA**	237595.8	12.9	237761.4	12.0	240155.4	11.4	258148.2	11.2	973660.8	11.8
	**TAS**	25416.0	1.4	26887.2	1.4	24544.8	1.2	28977.0	1.3	105825.0	1.3
	**NT**	19104.0		19280.4	1.0	19768.2	0.9	21818.4	1.0	79971.0	1.0
	**ACT**	43836.0	2.4	46851.0	2.4	50138.4	2.4	55332.6	2.4	196158.0	2.4
	**Australia**	1842715.2	22.4	1980960.6	24.1	2114805.2	25.7	**2298925.2**	**27.9**	8237406.2	100.0
**People with weekly income aboveAUD$2500**	**NSW**	116068.8	24.4	305027.4	37.0	495413.8	37.9	695794.8	35.7	1612304.8	35.4
	**VIC**	90461.0	19.0	188228.4	22.9	316388.8	24.2	470608.8	24.1	1065687.0	23.4
	**QLD**	225833.6	47.5	219874.8	26.7	219940.8	16.8	340370.4	17.5	1006019.6	22.1
	**SA**	11428.8	2.4	39818.4	4.8	69457.4	5.3	107842.2	5.5	228546.8	5.0
	**WA**	18221.3	3.8	31075.0	3.8	138384.0	10.6	232818.0	11.9	420498.3	9.2
	**TAS**	2781.5	0.6	8336.4	1.0	15098.8	1.2	24631.8	1.3	50848.5	1.1
	**NT**	1826.9	0.4	5748.0	0.7	10519.2	0.8	17251.8	0.9	35345.9	0.8
	**ACT**	8613.0	1.8	25425.0	3.1	42381.6	3.2	60350.4	3.1	136770.0	3.0
	**Australia**	475234.9	10.4	823533.4	18.1	1307584.4	28.7	**1949668.2**	**42.8**	4556020.9	100.0

Table 3 shows the percentage of primary land use types across different states of Australia during 1999–2010. A decreasing trend of production from natural environments was observed.

**Table 3:** Percentage of primary land use types across different states/territories of Australia during 1999–2010.

Primary Land use Types	Time Period	State/Territory							
		NSW	SA	TAS	VIC	WA	QLD	ACT	NT
**Conservation and natural environments**	1998–99	11.22	37.07	47.52	18.22	51.33	11.34	56.26	45.59
	2001–02	12.28	37.84	50.88	18.75	51.74	11.23	56.43	46.66
	2005–06	13.86	40.46	47.23	21.38	53.98	11.19	59.44	48.75
	2010–11	13.32	42.33	47.91	21.22	55.05	12.25	64.10	53.09
**Intensive uses**	1998–99	0.77	0.31	0.87	2.02	0.08	0.32	15.75	0.05
	2001–02	0.82	0.31	0.86	1.89	0.09	0.33	13.82	0.04
	2005–06	0.99	0.19	0.89	2.86	0.12	0.51	10.39	0.06
	2010–11	1.02	0.19	2.60	4.42	0.11	0.42	10.68	0.06
**Production from dryland agriculture and plantations**	1998–99	13.90	6.79	15.66	36.19	5.61	3.80	15.46	0.05
	2001–02	14.83	6.76	15.60	36.28	5.42	3.63	16.58	0.07
	2005–06	35.60	11.67	22.28	55.92	7.07	16.80	24.90	0.12
	2010–11	36.59	11.50	20.95	54.78	6.97	17.25	18.78	0.26
**Production from irrigated agriculture and plantations**	1998–99	1.35	0.17	1.13	2.91	0.02	0.29	0.05	0.00
	2001–02	1.64	0.19	1.34	2.90	0.02	0.35	0.29	0.01
	2005–06	1.19	0.18	1.28	3.13	0.02	0.30	0.02	0.00
	2010–11	0.84	0.17	1.12	2.15	0.03	0.25	0.13	0.00
**Production from relatively natural environments**	**1998–99**	**71.50**	**51.70**	**31.82**	**39.08**	**41.20**	**83.18**	**12.36**	**52.96**
	**2001–02**	**69.17**	**50.94**	**28.38**	**38.61**	**40.98**	**83.39**	**12.78**	**51.86**
	**2005–06**	**47.11**	**43.57**	**24.91**	**15.14**	**37.06**	**70.29**	**5.13**	**49.94**
	**2010–11**	**46.98**	**41.88**	**24.10**	**15.94**	**36.12**	**68.93**	**6.19**	**45.46**
**Water**	1998–99	1.23	3.87	2.05	1.34	1.65	0.96	0.11	1.25
	2001–02	1.23	3.88	2.05	1.34	1.65	0.96	0.11	1.25
	2005–06	1.21	3.84	2.29	1.33	1.63	0.77	0.12	1.00
	2010–11	1.21	3.84	2.30	1.33	1.63	0.78	0.12	1.00

Table 4 shows the descriptive statistics for annual climatic conditions during the period 1999–2010 and short-term future projections for 2020–2039 in capital cities of Australia. During 1999–2010, the highest average annual mean temperature was observed in Darwin (27.70°C) and lowest in Canberra (8.86°C). Similarly, the highest average annual mean temperature was projected for Darwin (32.96°C) and lowest for Canberra (12.24°C) for 2020–2039. Except Hobart and Canberra, the average annual mean temperature across different states is quite similar with a difference of 1–3°C during 1999–2010. The highest amount of annual rainfall was observed in Darwin (1780.94mm) and the lowest in Adelaide (537.57mm).

**Table 4:** Descriptive statistics of past (1999–2010) and future short term projection (2020–2039) of annual climatic conditions in capital cities of Australia.

Climatic variables	Capital cities (State/Territory)	Mean	Sd	Max	Range	Projected Climatic conditions (RCP 4.5, CESM1-CAM5 2020–39)
**Mean Temperature (°C)**	Sydney (NSW)	18.78	0.29	19.10	0.90	23.19
	Melbourne (VIC)	20.52	0.43	21.40	1.30	21.13
	Brisbane (QLD)	20.45	0.30	20.90	0.90	25.97
	Adelaide (SA)	17.60	0.40	18.35	1.20	23.1
	Perth (WA)	18.64	0.37	19.30	1.35	25.83
	Hobart (TAS)	13.28	0.33	13.65	1.05	15.58
	Darwin (NT)	27.70	0.38	28.25	1.15	32.96
	Canberra (ACT)	8.86	0.38	9.65	1.35	12.24
**Rainfall (mm)**	Sydney (NSW)	1100.68	240.35	1499.20	683.20	**Rainfall projection not available**
	Melbourne (VIC)	612.74	103.71	779.00	280.70	
	Brisbane (QLD)	1045.55	359.21	1728.80	1116.60	
	Adelaide (SA)	537.57	128.51	716.00	428.40	
	Perth (WA)	675.85	122.17	828.20	348.60	
	Hobart (TAS)	553.08	144.31	865.00	518.40	
	Darwin (NT)	1780.94	309.13	2257.20	1042.80	
	Canberra (ACT)	1148.34	221.29	1389.80	823.20	

### Temporal trend analysis

**Fig 1:**
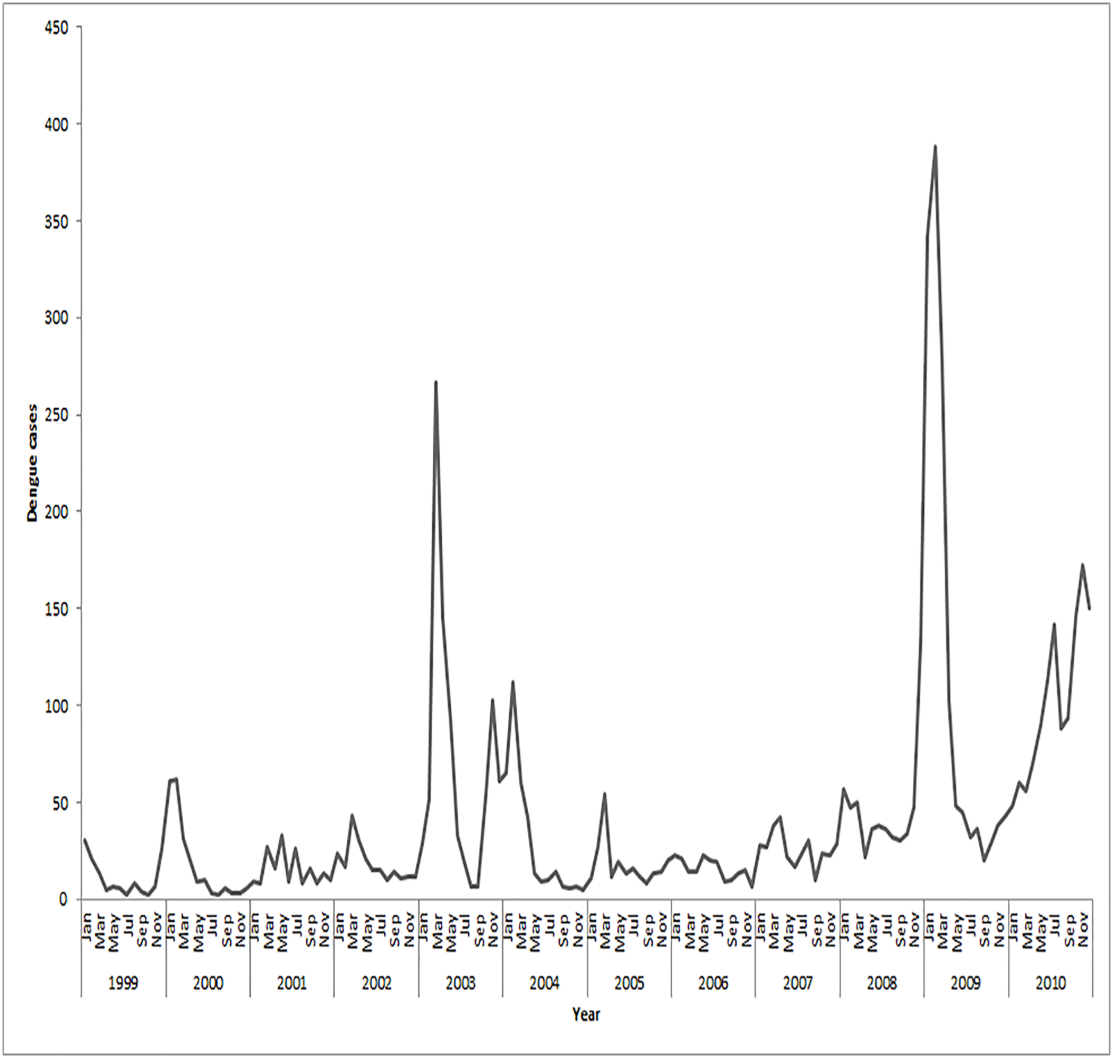
Monthly temporal pattern of dengue cases in Australia from 1999–2010 (Source: NNDSS).

Fig 1 displays the monthly epidemic patterns of dengue cases in Australia during 1999–2010. The largest epidemic was in 2009 with 388 cases across Australia. The temporal analysis clearly shows the seasonal variation of dengue cases and the dengue outbreaks mostly occurred during November to April of the year which could be explained by heavy rainfall in summer (December- February) and overseas visits of the most of the Australian residents during Christmas and summer school holidays.

**Fig 2:**
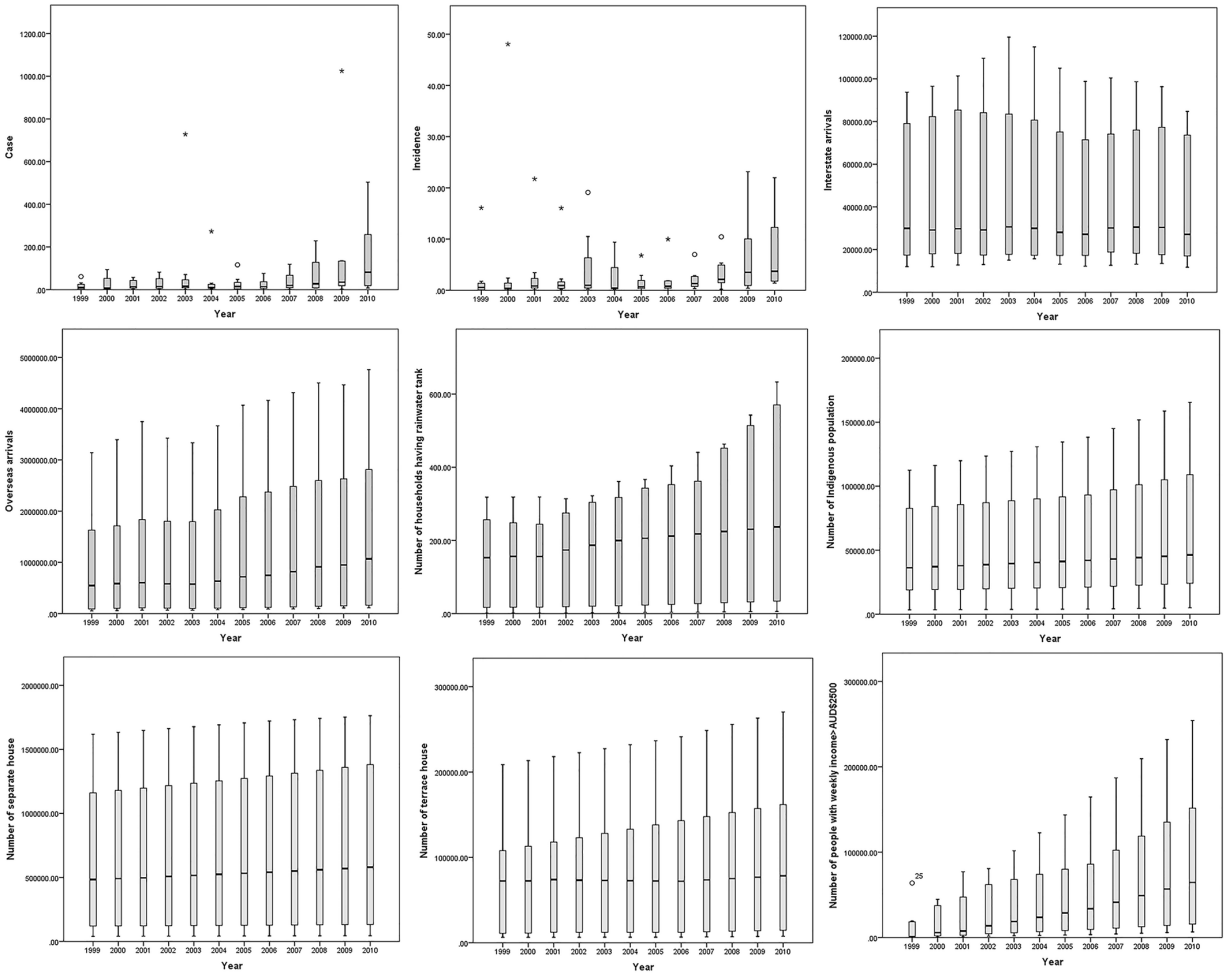
Box and whisker plot showing the temporal pattern of dengue and socio-demographic factors in Australia during 1999–2010.

Fig 2 shows an upward trend in the temporal distribution of dengue incidence and potential socio-demographic factors such as households having rainwater tanks, overseas arrivals, Indigenous populations and terrace houses.

Table 4 shows remarkable differences among the temperature changes during historic and projected periods. The projected temperature was highest for Darwin, followed by Brisbane. However, projected rainfall data is unavailable for the study areas.

Time series linear regression analyses results indicate that there was a significant increase of dengue and potential socio-demographic factors such as households having rainwater tanks, overseas arrivals, Indigenous populations and terrace house and economically advantage people. However, per year increasing trend of dengue in QLD was not statistically significant, which might be due to major outbreaks in 2003 and 2009 in this state. Per year increasing rate of dengue in QLD (β = 35.92) is one and half times higher compared to NSW (β = 14.67). NSW had highest increasing rate of overseas arrivals (β = 140.17) and terrace house types (β = 5.49). However, for Indigenous populations and households having rainwater tanks, QLD had the highest increasing trend of Indigenous people (β = 5529.4) and rainwater tanks (β = 31.6) compared to subsequent comparator NSW (Table 5).

**Table 5:** Summary statistics of temporal trend analysis (coefficient and R square) for socio- demographic and ecological variables, (p<0.05).

		NSW β (*R*^2^)	V IC β (*R*^2^)	QLD β (*R*^2^)	SA β (*R*^2^)	WA β (*R*^2^)	TAS β (*R*^2^)	NT β (*R*^2^)	ACT β (*R*^2^)
**Socio-demographic**	Dengue	**114.67** (0.67)*	**5.88** (0.41)*	**35.92** (0.18)	**1.92** (0.58)*	**25.32** (0.42)*	**0.45** (0.48)*	-2.33 (0.15)	**0.89** (0.35)*
	Interstate arrivals	-1121.2 (0.58)*	-714.1 (0.39)*	-830.6 (0.10)	-811.10 (0.81)*	**644.82** (1)*	-12.09 (0.00)	**77.10** (0.15)	-82.18 (0.11)
	Overseas arrivals (‘000)	**140.17** (0.89)*	**128.45** (0.96)*	**95.20** (0.92)*	**21.53** (0.90)*	**69.36** (0.88)*	**5.33** (0.96)*	**2.61**(0.42)*	**8.75** (0.97)*
	Rainwater tank (‘000)	**26.31** (0.94)*	**31.98** (0.83)*	**31.66** (0.86)*	**1.98** (0.40)*	**5.76** (0.99)*	**1.69** (0.98)*	**0.40** (0.87)*	**1.59** (0.87)*
	Indigenous population (‘000)	**4.66** (0.98)*	**1.19** (0.99)*	**3.83** (1)*	**0.60** (0.96)*	**0.73** (0.68)*	**0.30** (0.92)*	**0.61**(0.99)*	**0.13** (0.92)*
	Separate house (‘000)	**13.36** (0.99)*	**18.05** (0.99)*	**21.80** (1)*	**5.50** (1)*	**11.84** (0.99)*	**1.48** (0.99)*	**0.51** (0.99)*	**0.91** (0.99)*
	Terrace house (‘000)	**5.49** (0.99)*	**5.37** (0.99)*	**4.45** (0.99)*	**0.09** (0.80)*	**0.72** (0.67)*	**0.11**(0.22)	**0.10** (0.76)*	**0.42** (0.98)*
	People with weekly income above AUD$2500 (‘000)	**21.44** (1)*	**14.08** (0.98)*	**3.94** (0.53)*	**3.55** (0.99)*	**7.89** (0.98)*	**0.80** (0.98)*	**0.57** (0.98)*	**1.91** (0.99)*
**Land use types**	Conservation and natural environment	**0.79** (0.76)	**1.16** (0.83)	**0.27** (0.47)	**1.84** (0.96)*	**1.34** (0.94)*	-0.25 (0.03)	**2.46** (0.92)*	**2.65** (0.92)
	Intensive uses	**0.09** (0.92)	**0.82** (0.82)	**0.05** (0.48)	**0.05** (0.8)	**0.01** (0.72)	**0.52** (0.61)	**0.005** (0.45)	-1.86 (0.87)
	Production from dryland agriculture and plantations	**8.88** (0.83)	**7.54** (0.78)	**5.35** (0.81)	**1.90** (0.78)	**0.57** (0.71)	**2.26** (0.69)	**0.07** (0.86)	**1.83** (0.31)
	Production from irrigated agriculture and plantations	-0.19 (0.58)	-0.20 (0.38)	**0.02** (0.28)	-0.001(0.02)	**0.003** (0.6)	-0.009 (0.11)	**0.39** (0.87)	-0.003 (0.00)
	Production from natural environments	-9.56 (0.83)	-9.29 (0.79)	**5.59** (0.83)	**10.76** (0.58)8	-1.92 (0.89)*	-2.66 (0.94)*	-2.44 (0.91)*	-2.62 (0.71)
	Water	-0.008 (0.8)	-0.00 (0.8)	-0.07 (0.77)	**0.10** (0.82)	-0.01(0.8)	-0.00 (0.8)	-0.1(0.8)	0.00 (0.8)

* = significant; bold in colour showed increasing trend.

Among six primary land use types, only intensive uses, and production from dryland agriculture and plantations showed an overall increasing trend across different states of Australia. However, changes are not statistically significant (p>0.05) apart from production from relatively natural environments, and conservation and natural environments land use types in some states (Table 5).

### Spatial variation

**Fig 3:**
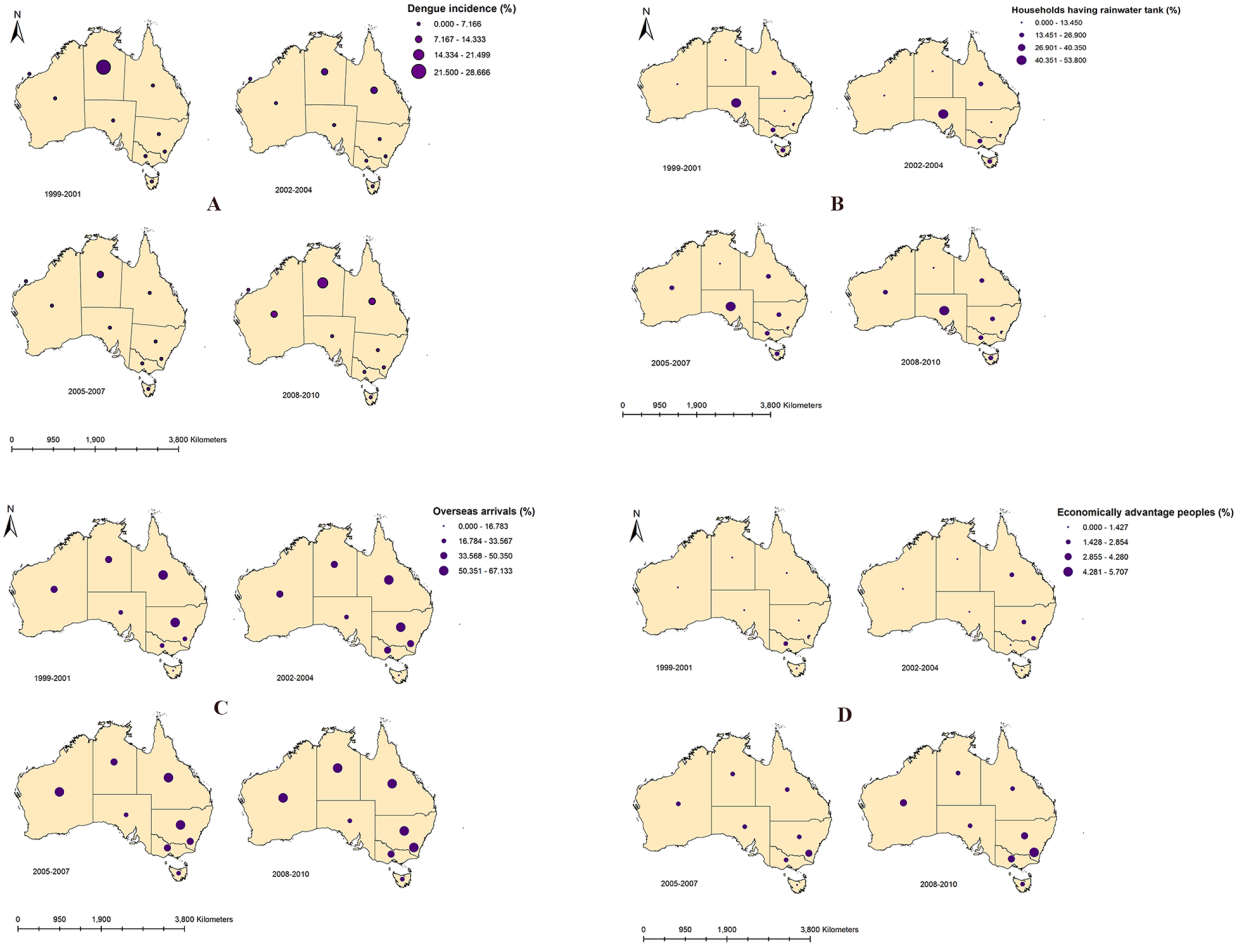
Maps showing the spatial variation of socio-demographic and ecological factors over different time periods (1999–2001; 2002–2004; 2005–2007; 2008–2010) across Australia. A. dengue incidence; B. households having rainwater tanks; C. overseas arrivals; D. economically advantage people.

Fig 3 depicts the spatial patterns of dengue, socio-demographic and ecological factors in four different time periods during 1999–2010. Over the study period, incidence rates were high in NT. QLD and NSW had highest percentage of overseas arrivals and spatial distribution illustrates the overall expansion of overseas arrivals to other states. Percentage of households with rainwater tanks showed spatial variation during the study period with highest percentage of rainwater tanks in SA. Highest percentage of economically advantage peoples showed geographical expansion. Terrace house types also showed spatial variation with highest percentage in SA over the study period (Fig A in S1 Fig). Spatial variation of proportion of separate houses (Fig B in S1 Fig), interstate arrivals (Fig C in S1 Fig), and Indigenous populations (Fig D in S1 Fig) remains static over the four different time periods. However, spatio-temporal variation of land use types across states is difficult to explain because of insignificant changes of land use types (S2 Fig).

## Discussion

This study has demonstrated the spatial, temporal trend of dengue and its potential socio-demographic and ecological determinants across Australia. Overall, an increasing trend of dengue and potential socio-demographic factors such as overseas arrivals, number of households having rainwater tanks and house types were observed across Australia.

Overseas and interstate arrivals are important factors in disseminating dengue in Australia. In Australia, dengue outbreaks initiate through the mosquito bites that transmits dengue virus, which was imported by infected travellers from dengue endemic countries [41]. Our results indicated an overall increasing trend of overseas arrivals across Australia with highest number of overseas arrivals in NSW followed by QLD (Table 1).

Travels including illegal shipping, cargo vessels and refugee boats not only increase the chance of virus distribution to other non-exposed states but also increase the risk of exotic mosquito importation into Australia. Even with upgraded inspection protocol placed in different ports, many ports in Australia, including Darwin, are not compliant. As a result, several interception and incursion of *A*. *aegypti* mosquitoes were reported from NT; 16 interceptions of exotic mosquitoes (*A*. *aegypti* and *A*. *albopictus*) in Darwin during 1998–2000; incursion of *A*. *aegypti* in 2004 in Tenant Creek, and in Groote Eylandt in 2006 [47]. Most recently, numerous incursions of *A*. *aegypti* occurred into towns of NT [48]. Although, all of the *Aedes* species were eliminated after intensive property inspections and elimination campaigns, mosquito could re-emerge in this place because of the extreme survival capacity of *Aedes* eggs, which can even be survived without water for several months [49]. Under these circumstances, it is not impractical to think that what happened to the vector of Ross River Virus [47], could happen for dengue. Therefore, upgraded inspection and surveillance should be placed in different ports across Australia with special emphasize in QLD because of local transmission and in NT due to frequent occurrence of *Aedes* mosquito in recent times. This may help to prevent the re-establishment of *A*. *aegypti* mosquito to other parts of Australia especially, NSW, NT and other parts of Australia.

During the study period, the number of households having rainwater tanks also showed an increasing trend across Australia which could increase the dengue transmission under projected climate change as evidence suggested that water vessels provide the breeding sites for mosquitoes [50–53]. According to future climate projection for Australia for the period 2020–2039 (Table 4), all the states and Territories except TAS and ACT will have favourable temperature (>16°C) for dengue virus transmission [54]. To adapt with changed climate as well as legislation due to water scarcity, installation of rainwater tanks could be increased in future which could possibly be supportive factors for dengue transmission. Currently, the trend of prolific water storage is similar to earlier years (1904–1943) when *A*. *aegypti* was present and dengue epidemics were common in Brisbane [55]. Even though recently installed rainwater tanks maintain the Australian Standards (Queensland Public Health Act 2005 and the Public Health Regulation 2005, however, 50 out of 807 rainwater tanks inspected in Brisbane are found to be non-compliant with Australian legislation [55] and over time more rainwater tanks may become defective, as evidenced elsewhere [56]. Evidence from the literature has suggested different housing types are important predictors of dengue transmission [30, 31, 57]. From this study, it is clear that terrace houses are most commonly found in NSW, VIC and QLD and followed an increasing trend. This terrace house could potentially increase the future dengue risk in these states because of its unique design, capability to store water which is favourable for mosquito breeding [31].

Land use or land cover changes can potentially affect human health in relation to dengue by influencing the mosquito’s habitat [29]. Several researchers have found significant relationship with dengue transmission and land use or land cover types [26–29, 58, 59]. Land cover such as residential area per capita, construction area, shrubs, grassland (wet), water area, and cropland (paddy field) have been found as significant predictors of dengue transmission [59]. In this study, only intensive uses, and production from dryland agriculture and plantations showed an overall increasing trend. However, further study regarding land use types and their impacts on dengue infections is required.

Socio-economic factors are also responsible for variation of dengue transmission [17, 34, 35]. Hu et al., [17] have used Socio-economic Index for Areas (SEIFA) in Australia and found that for each unit increase of SEIFA, overseas acquired cases of dengue were increased by 1%. It is explained that economically rich people could have more chance to travel overseas and outdoor activities which in turn have increased the chance of exposure to mosquito bites and thereby increasing the chances of acquiring dengue. In support of this, our study has also found that NSW has higher number of overseas acquired dengue cases which could be due to higher number of economically advantaged people (Table 1).

From the study, it is appeared that potential socio-ecological factors followed an increasing trend and highest number of all of these factors, except interstate visitors, was higher in NSW. Interstate visitors are highest in QLD where dengue mosquito *A*. *aegypti* are present and every year frequent outbreaks occur in this region. Geographically, NSW is close to QLD. Climate change, movement of population, urbanisation, and transportation system aids the establishment and expansion of geographical range of *Aedes* mosquito [21, 39]. This may result in expansion of dengue mosquito to other parts of Australia especially NSW where climates are favourable and potential socio-ecological factors are already present to provide the ecological niche for mosquito. This may in turn increase the occurrence of dengue cases with local transmission in near future owing to trends toward increased terrace houses, upgraded socio-economic status, urbanisation, water tank installation and, possibly, environmental change.

This is the first study that demonstrated the spatial and temporal trend of dengue and potential socio-demographic and ecological factors at state level in Australia; further indicated the probable future trend of dengue under projected climate change, consequent socio- demographic and ecological changes. The increasing trend of socio- demographic and ecological factors such as overseas arrivals, households having rainwater tank, increased number of economically sufficient people may pose a future threat on local transmission of dengue in other states previously unoccupied by *Aedes* mosquitoes. This study is an impetus for future investigation of socio-demographic and ecological impacts on dengue in a comprehensive manner. It will improve the accuracy of the predictive model for developing early warning systems; and hence improve the existing surveillance and disease prevention efforts.

This study has some limitations. As an ecological study, this study is subject to measurement and information biases. For example, underreporting is common as asymptomatic dengue patient never seek medical attention. Use of aggregated data at large spatial scale (state level) could lead to biasness in terms of variation in space. In this study, primary land use types, a broad category, was used to obtain an insight of the changes over time. However, our future study is aiming to explore in detail the land use types and their relationship with dengue at Local Government Area level in Queensland depending on quality and availability of the data. It is not clear whether the most cases are coming from urbanised area, therefore further analysis at small spatial scale with more specific land use types such as residential area or agricultural areas are required.

In conclusion, this study illustrated the spatio-temporal trend of dengue, potential socio-demographic and ecological factors across Australia. Spatial variation and increased temporal trend of socio-ecological factors under projected increased temperature may pose a future threat on local transmission of dengue across Australia if distribution of *Aedes* mosquitoes expanded to other parts of Australia under changed climate and owing to availability of socio-ecological factors. Overall, this study shows the direction and importance of placing upgraded mosquito surveillance at different ports to reduce the chance of vector mosquitoes being imported all over Australia.

## Supporting information

S1 Fig Maps showing the spatial variation of socio-demographic and ecological factors over different time periods (1999–2001; 2002–2004; 2005–2007; 2008–2010) across Australia. A. Terrace house; B. separate house; C. interstate arrivals; D. Indigenous populations.
(TIF)

S2 Fig Maps showing the spatial variation of land use change. (TIF)

S1 Table Summary statistics of temporal trend analysis for socio-demographic factors. (DOCX)

S2 Table Summary statistics of temporal trend analysis for land use types. (DOCX)
