# Supplementary figures and images for: Socio-demographic, ecological factors and dengue infection trends in Australia
Source: PLoS One. 2017 Oct 2;12(10):e0185551. doi: 10.1371/journal.pone.0185551 (PMC5624700; doi:10.1371/journal.pone.0185551)

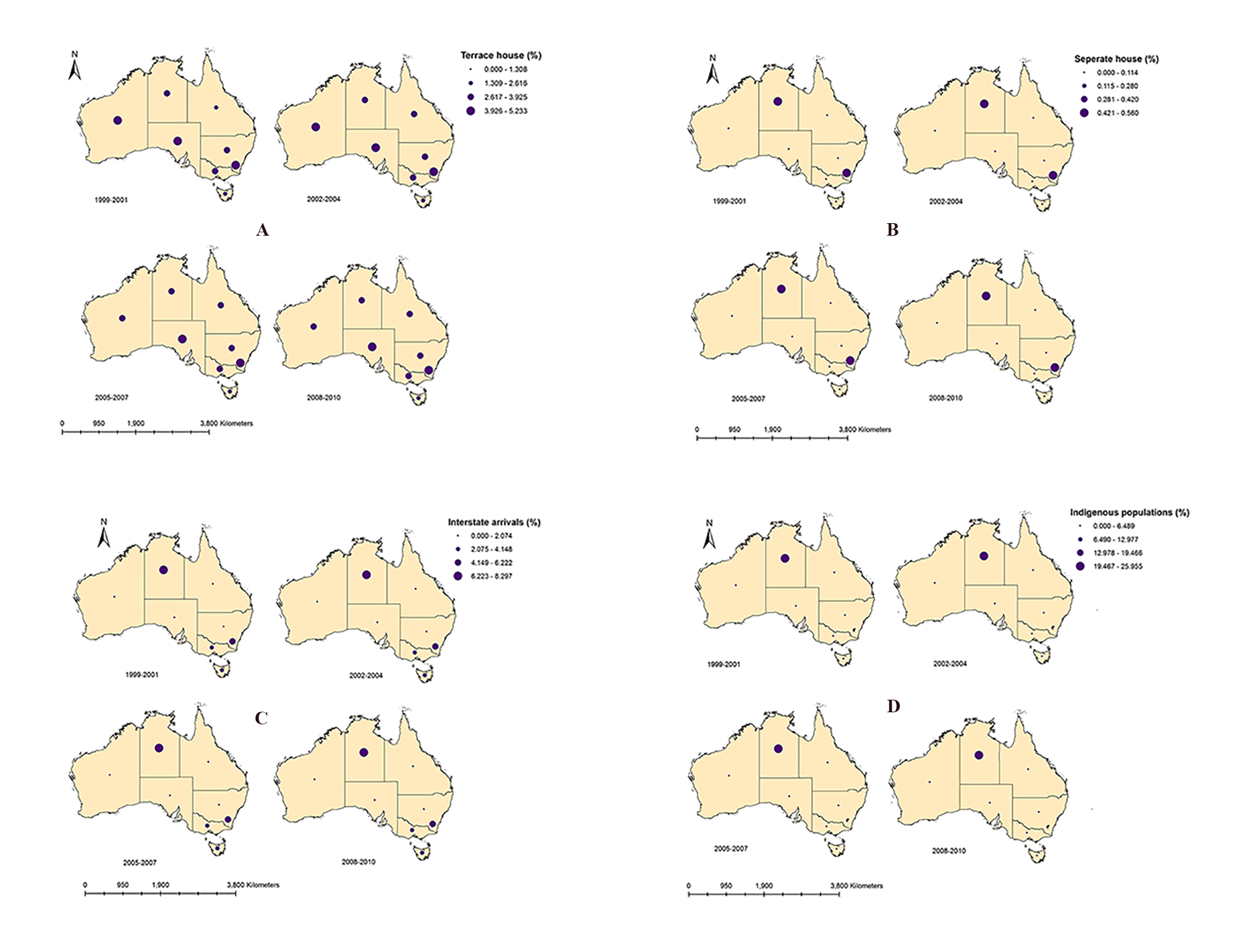

Supplement: S1 Fig — A. Terrace house; B. separate house; C. interstate arrivals; D. Indigenous populations. (TIF) [file pone.0185551.s001.tif]

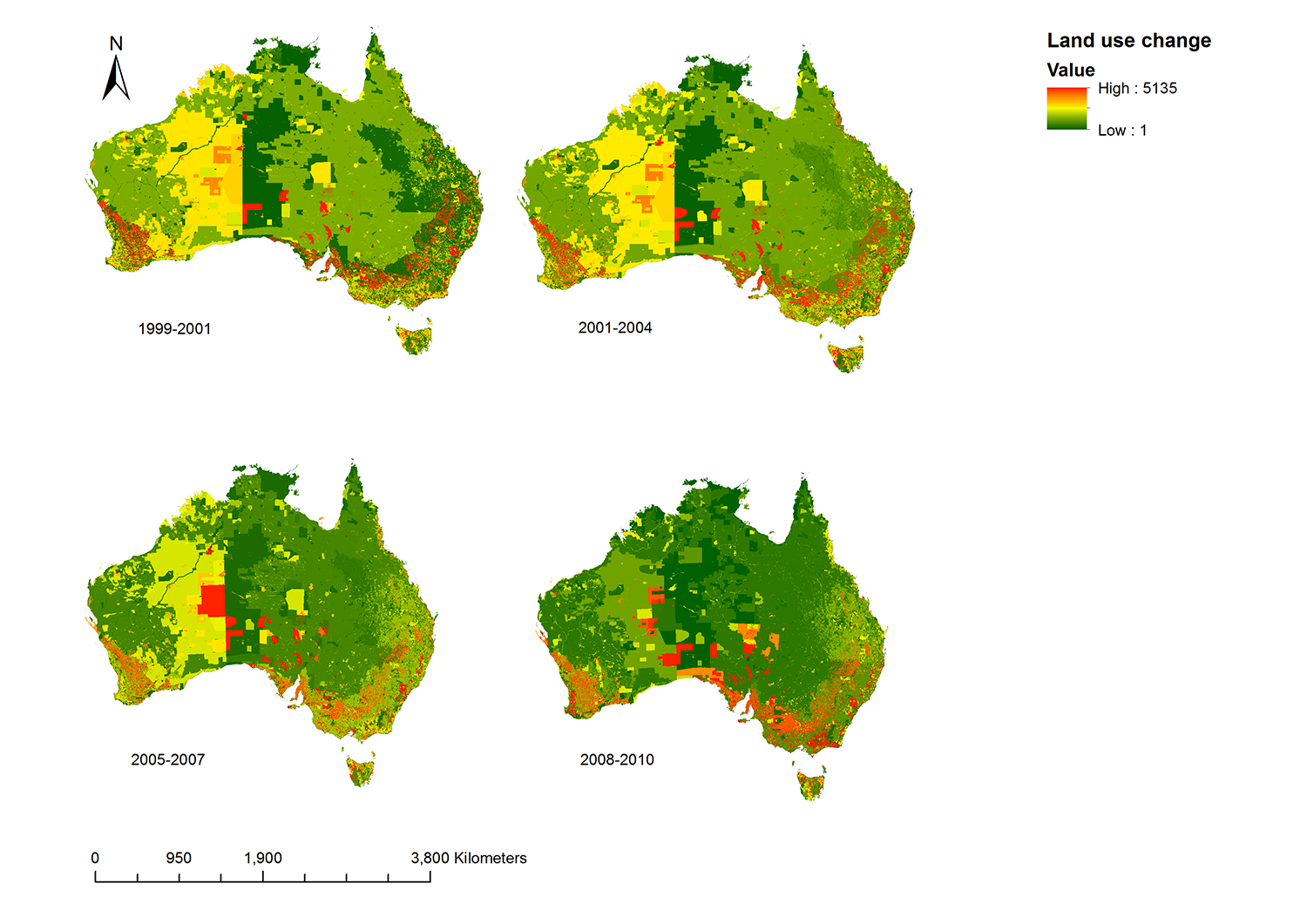

Supplement: S2 Fig — (TIF) [file pone.0185551.s002.tif]
