# Supplementary material for: Socio-demographic, ecological factors and dengue infection trends in Australia
Source: PLoS One. 2017 Oct 2;12(10):e0185551. doi: 10.1371/journal.pone.0185551 (PMC5624700; doi:10.1371/journal.pone.0185551)
Supplement: S1 Table — (DOCX) [file pone.0185551.s003.docx]

**S1 Table. Summary statistics of temporal trend analysis for socio-demographic factors.**

| Variables |  | States/Territories | | | | | | | |
| --- | --- | --- | --- | --- | --- | --- | --- | --- | --- |
|  |  | **NSW** | **VIC** | **QLD** | **SA** | **WA** | **TAS** | **NT** | **ACT** |
| Dengue | β (C.I) | 14.67 (7.4-21.95) | 5.88 (0.93-10.83) | 35.92 ( -18.25-90.09) | 1.92 (0.78-3.07) | 25.32 (4.41-46.22) | 0.45 (0.12-0.78) | -2.33 (-6.23-1.57) | 0.89 (0.04-1,74) |
|  | $R^{2}$ (p) | 0.67 (0.00) | 0.41 (0.03) | 0.18 (0.17) | 0.58 (0.00) | 0.42 (0.02) | 0.48 (0.02) | 0.15 (0.21) | 0.35 (0.04) |
| Interstate arrivals | β (C.I) | -1121.2 (-1760-06-446.47) | -714.1 (-1338.78- -90.01) | -830.6 (-2894.74-933.46) | -811.10 (-1084.49-537.71) | 644.82 (215.37-1074.29) | -12.09 (-304.00-279.82) | 77.10 (-54.29-208.49) | -82.18 (-248.63-84.28) |
|  | $R^{2}$(p) | 0.58 (0.00) | 0.39 (0.03) | 0.10 (0.3) | 0.81 (0.00) | 1 (0.01) | 0.00 (.93) | 0.15 (0.22) | 0.11 (0.30) |
| Overseas arrivals | β (C.I) | 140176 (105997.49-174355.21) | 128454.87 (110649.82-146259.93) | 95208.03 (7591.34-114498.71) | 21534.36 (16715.88-26352.85) | 69366.57 (50977.49-87755.66) | 5335.44 (4599.56-6071.33) | 2611.8 (443.56-4780.05) | 8746.25 (7680.67-9811.84) |
|  | $R^{2}$ (p) | 0.89 (0.00) | 0.96 (0.00) | 0.92 (0.00) | 0.90 (0.00) | 0.88 (0.00) | 0.96 (0.00) | 0.42 (0.02) | 0.97 (0.00) |
| Rainwater tank | β (C.I) | 26.31 (21.63-30.98) | 31.98 (21.85-42.10) | 31.66 (22.67-40.66) | 1.98 (0.28-3.68) | 5.76 (5.42-6.10) | 1.69 (1.56-1.83) | 0.40 (0.29-0.51) | 1.59 (1.16-2.04) |
|  | $R^{2}$ (p) | 0.94 (0.00) | 0.83 (0.00) | 0.86 (0.00) | 0.40 (0.03) | 0.99 (0.00) | 0.98 (0.00) | 0.87 (0.00) | 0.87 (0.00) |
| Indigenous population | β (C.I) | 4658.98 (4148.68-5169.28) | 1193.16 (1101.67-1284.66) | 3827.27 (3389.87-4264.67) | 598.73 (511.56-685.90) | 732.30 (377.68-1086.92) | 295.92 (235.23-356.62) | 605.58 (594.18-616.98) | 133.5 (104.93-162.07) |
|  | $R^{2}$ (p) | 0.98 (0.00) | 0.99 (0.00) | 1 (0.00) | 0.96 (0.00) | 0.68 (0.00) | 0.92 (0.00) | 0.99 (0.00) | 0.92 (0.00) |
| Separate house | β (C.I) | 13366.1 (12665.90-14076.32) | 18050.92 (16867.84-19234.02) | 21801.21 (21774.54-21827.88) | 5505.8 (5488.90-5522.74) | 11841.05 (11354.41-123227.69) | 1478.18 (1418.58-1537.78) | 499.75 (467.30-532.29) | 896.8 (890.44-903.16) |
|  | $R^{2}$ (p) | 0.99 (0.00) | 0.99 (0.00) | 1 (0.00) | 1 (0.00) | 0.99 (0.00) | 0.99 (0.00) | 0.99 (0.00) | 0.99 (0.00) |
| Terrace house | β (C.I) | 5489.8 (5069.86-5909.60) | 5367.08 (5173.63-5560.53) | 4452.36 (4371.85-4532.88) | 91.95 (-128.25-312.16) | 719.65 (359.92-1079.36) | 105.22 (-34.25-244.70) | 96.45 (58.85-134.06) | 420.72 (376.75-464.69) |
|  | $R^{2}$ (p) | 0.99 (0.00) | 0.99 (0.00) | 0.99 (0.00) | 0.80 (0.4) | 0.67 (0.00) | 0.22 (0.12) | 0.76 (0.00) | 0.98 (0.00) |
| People with weekly income above AUD$2500 | β (C.I) | 21444.71 (21214.22-21675.92) | 14075.74 (12858.65-15292.84) | 3939.04 (1327.08-6551.00) | 3547.56 (3345.88-3749.25) | 7888.9 (7095.22-8682.59) | 803.89 (724.70-883.07) | 567.91 (511.73-624.09) | 1913.5 (1890-1936.85) |
|  | $R^{2}$ (p) | 1 (0.00) | 0.98 (0.00) | 0.53 (0.01) | 0.99 (0.00) | 0.98 (0.00) | 0.98 (0.00) | 0.98 (0.00) | 0.99 (0.00) |

Β, Beta coe-efficient; C.I, 95% Confidence Interval; $R^{2}$, R –square; p, p values
