# Supplementary material for: Socio-demographic, ecological factors and dengue infection trends in Australia
Source: PLoS One. 2017 Oct 2;12(10):e0185551. doi: 10.1371/journal.pone.0185551 (PMC5624700; doi:10.1371/journal.pone.0185551)
Supplement: S2 Table — (DOCX) [file pone.0185551.s004.docx]

**S2 Table. Summary statistics of temporal trend analysis for land use types.**

| Variables |  | States/Territories | | | | | | | |
| --- | --- | --- | --- | --- | --- | --- | --- | --- | --- |
|  |  | **NSW** | **VIC** | **QLD** | **SA** | **WA** | **TAS** | **NT** | **ACT** |
| Conservation and natural environment | β (C.I) | 0.79(-0.5- -2.1) | 1.16 (-0.40-2.72) | 0.27 (0.59-1.12) | 1.84 (0.75-2.92) | 1.34 (0.32-2.36) | -0.25(-4.10-3.66) | 2.46 (0.20-4.72) | 2.65 (-0.42-5.73) |
|  | $R^{2}$(p) | 0.76 (0.12) | 0.83 (0.09) | 0.48 (0.30) | 0.96 (0.02) | 0.94 (0.03) | 0.03 (0.81) | 0.92 (0.04) | 0.92 (0.07) |
| Intensive uses | β (C.I) | 0.09 (0.01-0.17) | 0.82 (-0.34-1.98) | 0.05 (-0.10-0.20) | 0.05 (-0.12-0.02) | 0.01 (-0.01-0.03) | 0.52 (-0.75-1.79) | 0.01 (-0.01-0.02) | -1.86 (-4.06-0.33) |
|  | $R^{2}$ (p) | 0.92(0.12) | 0.82 (0.09) | 0.49 (0.30) | 0.8 (0.10) | 0.72 (0.15) | 0.61 (0.22) | 0.46 (0.32 | 0.87 (0.07) |
| Production from dryland agriculture and plantations | β (C.I) | 8.88 (-3.17-20.93) | 7.54 (-4.76-19.84) | 5.35 (-2.59-13.29) | 1.90 (-1.15-4.95) | 0.57 (-0.52-1.67) | 2.26 (-2.32-6.82) | 0.07 (-0.02-0.15) | 1.83 (-6.39- 10.05) |
|  | $R^{2}$ (p) | 0.83(0.09) | 0.78 (0.12) | 0.81 (0.10) | 0.78 (0.12) | 0.71 (0.15) | 0.69 (0.17) | 0.86 (0.07) | 0.31 (0.56) |
| Production from irrigated agriculture and plantations | β (C.I) | -0.19 (-0.70-0.30) | -0.20 (0.99-0.59) | 0.02 (-0.10-0.06) | -0.00 (-0.02-0.02) | 0.00 (-0.00-0.01) | -0.01 | 0.39 (-0.01-0.01) | -0.00 (-0.29-0.29) |
|  | $R^{2}$ (p) | 0.59 (0.23) | 0.38 (0.38) | 0.29 (0.47) | 0.02 (0.87) | 0.6 (0.22) | 0.11 (0.89) | 0.87 (0.74) | 0.00 (0.97) |
| Production from natural environments | β (C.I) | -9.56 (-22.33-3.20) | -9.29(-23.69-5.12) | -5.59 (-13.28-2.11) | 10.76 (-7.48-0.11) | -1.92 (-4.00-0.17) | -2.66 (-4.6—0.69) | -2.44 (-4.80- -0.08) | -2.62 (-7.74- 2.51) |
|  | $R^{2}$ (p) | 0.83 (0.08) | 0.79 (0.11) | 0.83 (0.10) | 0.58 (0.05) | 0.89 (0.06) | 0.94 (0.03) | 0.91 (0.05) | 0.71 (0.16) |
| Water | β (C.I) | -0.008 (-0.02-0.00) | -0.00 (-0.01-0.00) | -0.07 (-0.19-0.05) | 0.10 (-0.04-0.15) | -0.00 (-0.02-0.04) | -0.10 (-0.04-0.24) | -0.1 (-0.25-0.05) | 0.00 (-0.00-0.01) |
|  | $R^{2}$ (p) | 0.8(0.11) | 0.8(0.11) | 0.77(0.12) | 0.82 (0.19) | 0.8 (0.11) | 0.82 (0.10) | 0.8 (0.11) | 0.8 (0.11) |

Β, Beta coe-efficient; C.I, 95% Confidence Interval; $R^{2}$, R –square; p values
